# Accelerated Cell Aging in Female APOE-ε4 Carriers: Implications for Hormone Therapy Use

**DOI:** 10.1371/journal.pone.0054713

**Published:** 2013-02-13

**Authors:** Emily G. Jacobs, Candyce Kroenke, Jue Lin, Elissa S. Epel, Heather A. Kenna, Elizabeth H. Blackburn, Natalie L. Rasgon

**Affiliations:** 1 Robert Wood Johnson Foundation Health and Society Scholars Program, Center for Health and Community, University of California San Francisco, San Francisco, California, United States of America; 2 Kaiser Permanente Division of Research, Oakland, California, United States of America; 3 Department of Biochemistry and Biophysics, University of California San Francisco, San Francisco, California, United States of America; 4 Department of Psychiatry, University of California San Francisco, San Francisco, California, United States of America; 5 Stanford Center for Neuroscience in Women's Health, Department of Psychiatry and Behavioral Sciences, Stanford University School of Medicine, Stanford, California, United States of America; University of the Witwatersrand, South Africa

## Abstract

Apolipoprotein-ε4 (APOE-ε4) is a major genetic risk factor for cognitive decline, Alzheimer's disease (AD) and early mortality. An accelerated rate of biological aging could contribute to this increased risk. Here, we determined whether APOE-ε4 status impacts leukocyte telomere length (TL) and the rate of cellular senescence in healthy mid-life women and, further, whether hormone replacement therapy (HT) modifies this association. Post-menopausal women (N = 63, Mean age = 57.7), all HT users for at least one year, were enrolled in a randomized longitudinal study. Half of the participants (N = 32) remained on their HT regimen and half (N = 31) went off HT for approximately two years (Mean  = 1.93 years). Participants included 24 APOE-ε4 carriers and 39 non-carrier controls. Leukocyte TL was measured at baseline and the end of year 2 using quantitative polymerase chain reaction. Logistic regression analysis indicated that the odds of an APOE-ε4 carrier exhibiting telomere shortening (versus maintenance/growth) over the 2-year study were more than 6 (OR  = 6.26, 95% CI  = 1.02, 38.49) times higher than a non-carrier, adjusting for established risk factors and potential confounds. Despite the high-functioning, healthy mid-life status of study participants, APOE-ε4 carriers had marked telomere attrition during the 2-year study window, the equivalent of approximately one decade of additional aging compared to non-carriers. Further analyses revealed a modulatory effect of hormone therapy on the association between APOE status and telomere attrition. APOE-ε4 carriers who went off their HT regimen exhibited TL shortening, as predicted for the at-risk population. APOE-ε4 carriers who remained on HT, however, did not exhibit comparable signs of cell aging. The opposite pattern was found in non-carriers. The results suggest that hormone use might buffer against accelerated cell aging in mid-life women at risk for dementia. Importantly, for non-carrier women there was no evidence that HT conferred protective effects on telomere dynamics.

## Introduction

Telomeres are DNA-protein complexes that cap the ends of eukaryotic chromosomes and protect against genomic instability. Telomere length reflects the replicative history of a cell (telomeres shorten with each cell division) and a cell's cumulative exposure to inflammation and oxidative stress [1]–[3]. Telomere loss can be counteracted by telomerase, which adds telomeric repeats to terminal DNA. Leukocyte telomere length (TL) has emerged as a putative index of cellular age that predicts the incidence of age-related diseases [4]–[10] as well as all-cause and disease-specific mortality in older adults [11]–[15].

Accumulating evidence suggests a link between TL and neurodegenerative diseases. Leukocyte TL predicts pre-clinical cognitive decline in the elderly [16], [17]; patients with Alzheimer's disease exhibit shorter TL cross-sectionally compared with healthy individuals [12]; and within AD patients, T cell telomere length correlates with disease severity [9], although some studies have not found such relationships [18], [19]. Recent evidence from a telomerase-deficient mouse model demonstrates the widespead consequences of telomere attrition on neurodegeneration, including reduced proliferation of neural progenitor cells, restricted neurogenesis, and atrophy of white matter tracts. Remarkably, these age-related degenerative phenotypes were reversed following reactivation of endogenous telomerase activity [20].

Apolipoprotein-ε4 (APOE-ε4) is a major genetic risk factor for AD [21], preclinical cognitive decline [22], [23] and early mortality [24]. Cross-sectional evidence shows that APOE-ε4 carriers have shorter leukocyte telomeres compared to non-carriers [12], [25]. These observations provide preliminary support for the proposal that APOE-ε4 carriers undergo premature cell aging relative to non-carriers, but direct longitudinal evidence tracking telomere attrition over time is lacking and is necessary for establishing a direct relationship between this genetic risk factor and cell aging.

In addition to its association with the APOE-ε4 risk allele, telomere dynamics show a striking sex difference. At birth TL is equivalent between the sexes, but by adulthood women have significantly longer age-adjusted TL than men [26], [27]. This sex difference in TL could be driven, in part, by circulating estrogen. Estrogen up-regulates telomerase activity [28]–[32] and may reduce oxidative stress [33], [34], two putative pathways that could buffer against telomere shortening. Further, telomerase exhibits cyclic changes in activity over the menstrual cycle [35], [36] and over the life-course greater endogenous estrogen exposure (estimated by the duration of reproductive years of life) is associated with longer TL in post-menopausal women [37]. What remains unknown is whether exogenous estrogen, such as estrogen replacement during menopause, confers a similar protective effect on cell aging. To date, the only study examining the *in vivo* relationship between HT and telomere length found that women who had been on HT (including 0.625 mg conjugated equine estrogen or 2 mg estradiol paired with progesterone) for more than five years had longer TL cross-sectionally compared to age-matched women who had not used HT [38]. However, critically, the authors noted that women who exercised regularly and took daily vitamins were better represented in the group of women using HT, which may confound the results as both of these factors have been reported to be associated with longer TL [39], [40]. To date, no randomized longitudinal study has examined the impact of HT use on cellular aging.

Given *in vitro* and *in vivo* evidence that estrogen and APOE may act synergistically [41]–[43], and evidence of sex differences in the association between APOE-ε4 carrier status and AD biomarkers [44]–[48], we were motivated to measure the impact of hormone replacement therapy on telomere dynamics as a function of APOE status. In the present study, we examined change in TL over a 2-year period in healthy, mid-life women who were either carriers of the APOE-ε4 risk allele or non-carrier controls. Using a longitudinal, randomized design we first tested whether ε4 carriers exhibit accelerated cell aging; we then examined whether hormone replacement buffers against TL decline. We hypothesized that APOE-ε4 carriers would undergo greater TL shortening over the two-year period compared to non-carriers and that hormone use, initiated at the onset of the menopausal transition, would protect against telomere shortening.

## Materials and Methods

### Study population and procedure

Participants for this investigation included 63 high-functioning post-menopausal women (mean age  = 57.72, SD  = 5.59). Participants were recruited through community flyers and advertisements and were selected to be at-risk for cognitive decline (including APOE-ε4 carriers and family history of AD) and stable HT users (defined as taking HT for 1 year or longer). Exclusion criteria included a history of neuropsychiatric disorder, history of drug or alcohol abuse, significant cognitive impairment (defined as any impairment in daily functions and/or MMSE <24), current depression and history of major medical illness. Data on reproductive history, including age at menarche and menopause, parity, use of hormonal contraception during reproductive years, timing of HT use in relation to the duration of perimenopause, and type of menopausal symptoms was collected by the study physician during the screening evaluation. During the initial screen a psychiatric, physical, and neurological examination was conducted, including clinical blood chemistries to confirm postmenopausal status and general medical health. Of the 80 participants that passed the initial screen, a final sample of 63 were enrolled in the longitudinal study and underwent HT randomization. All participants were Caucasian except 1 Asian American. The sample included 24 APOE-ε4 carriers (22 were single ε4 carriers; 2 were double ε4-ε4 carriers) and 39 non-carrier controls (including 35 ε3-ε3 carriers and 4 ε2-ε3 carriers). Of the 63 enrollees, 31 participants (mean age  = 57.31, SD  = 4.78) were randomized to go off HT and 32 participants (mean age  = 58.12, SD  = 4.42) were randomized to remain on HT for the duration of the 2-year study. Hormone therapy formulations varied across women, and are representative of the HT regimens commonly prescribed in a clinical setting. Formulations included estrogen-alone (n = 22) and estrogen+progesterone (E+P; n = 41). In terms of estrogen type, for estrogen-only users 36% were taking conjugated equine estrogen (CEE) and 64% were on estradiol. Similarly, among E+P users 32% were on CEE and 68% were on estradiol. Due to difficulty obtaining viable peripheral blood mononuclear cells from samples or a subject's refusal of the blood draw, Time 1 (baseline) TL was not obtained in 8 participants (Time 1 TL, n = 55) and Time 2 TL was obtained in n = 45. Thus, a sample size of n = 42 was available for examining the two-year change in TL, with 24 APOE non-carriers and 18 carriers, and the larger samples were available for assessing cross-sectional relationships between APOE genotype and TL. All participants gave written informed consent. The study was approved by the Institutional Review Board for Human Research at Stanford University.

### APOE genotyping

DNA extraction and analysis was conducted at Stanford University School of Medicine (Stanford, CA). Blood for genotyping (approximately 6cc) was drawn at the time of eligibility screening after informed consent. Genotyping for APOE-ε4 was performed according to the restriction isotyping protocol of Hixson and Vernier as previously reported in detail [49]. To ensure quality assurance, 20% of the samples were randomly chosen for repeated testing. These analyses showed 100% concordance across genotyped samples.

### Leukocyte telomere length measurement

Subjects underwent blood sampling using venipuncture in a fasting state during the morning hours between 7 am to 10 am. All samples were processed for isolation of mononuclear cells within 1 h of collection. One ml of cryopreserved peripheral blood mononuclear cells (PBMCs) was thawed at 37°C, washed twice with 10 ml of cold DPBS (Invitrogen, Calsbard, CA). Cell pellets were collected and DNA was prepared using a Puregene DNA purification Kit (QIAGEN, Valencia, CA).

Quantitative polymerase chain reaction (Q-PCR) was used to measure TL in the genomic DNA of peripheral leukocytes by determining the ratio of telomere repeat sequence copy number to a reference single copy gene copy number (T/S ratio) in each sample relative to a reference sample. The T and S values were each determined by the standard curve method using a serially diluted reference DNA and the T/S ratio was derived from the T and S value for each sample. Each T/S value was later converted to number of base pairs (bp). The conversion from T/S ratio to base pairs was calculated based on comparison of telomeric restriction fragment (TRF) length from Southern blot analysis and T/S ratios using DNA samples from the human cell line IMR90 at different population doublings. The slope of the linear regression line through a plot of T/S ratio (the x axis) versus mean TRF length (the y axis) is the number of base pairs of telomeric DNA corresponding to a single T/S unit. The formula to convert T/S ratio to base pairs was base pairs  = 3,274+2,413*(T/S). The inter-assay coefficient of variation (COV) for the baseline TL assay was 3.7% and the intra-assay COV was 2.5%. For Time 2 TL measurement the inter-assay COV was 4.5%, and the intra-assay COV was 2.5%. Results obtained with the Q-PCR method are strongly associated with the traditional terminal restriction fragment length index of TL obtained by Southern blot technique [50].

### Statistical analysis

**Figure 1:**
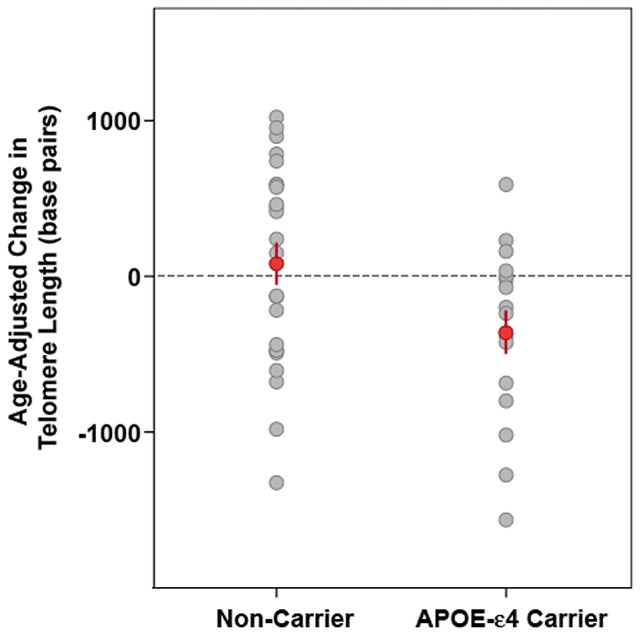
APOE-ε4 genotype and telomere attrition. Dot plot illustrating age-adjusted change in leukocyte telomere length (TL) in carriers of the APOE-ε4 risk allele and non-carrier controls. APOE-ε4 carriers had significantly greater age-adjusted LTL shortening over the 2 year study period than non-carrier control participants [*F*(1,39) 4.89, *p* = .03].

Logistic regression modeling and analysis of covariance (ANCOVA) were used to test our primary hypothesis regarding the association between APOE-ε4 carrier status and accelerated cell aging as well as the potential modifying role of hormone replacement therapy. The variables TL_Time1_ and TL_Time2_ were recorded as T/S ratios and converted to base pairs for description. Change in TL was computed as (TL_Time2_ – TL_Time1_). For logistic regression modeling, the outcome was computed as the odds of TL shortening (TL_Time2_ – TL_Time1_ <0) versus maintenance (≥0). Logistic regression analyses were used to examine the effect of APOE-ε4 carrier status on the odds of TL shortening over the 2 year study period, adjusting for factors previously found to be associated with TL and potential confounds, including age, education, HT group and baseline TL. Analysis of covariance was used to further examine the relationship between APOE-ε4 status (carrier, non carrier) and hormone group (randomization on, off) on TL change, adjusting for known confounds and study covariates (age, education, baseline TL and time between sampling). Given accumulating evidence that telomere shortening is proportional to baseline telomere length [51], [14] we adjusted for baseline TL in our longitudinal model of TL change. For the purpose of graphically representing our findings, age-adjusted change in TL for each participant was calculated as the observed TL change plus the difference between the mean and actual age multiplied by the unstandardized β obtained by regressing TL change on age (Figure 1). All analyses were performed using SPSS version 20.0.

## Results

Descriptive data for the sample are presented in Table 1. Participants randomized into hormone ‘ON’ and hormone ‘OFF’ groups did not differ significantly in age (range: 49–65; 50–69 years, respectively), years of education, body mass index (BMI) or baseline cognitive function. Hormonal variables, including age at menarche, age at menopause and number of years on HT at enrollment, did not differ between groups except in one case: women randomized off HT were more likely to have been on an HT regimen that included 17β-estradiol (versus conjugated equine estrogen).

**Table 1:** Characteristics of the study sample by HT randomization.

		OFF HT	ON HT	*p*
		(*n = 31*)	(*n = 32*)	
Age (yrs)		57.6 (4.78)	58.10 (4.49)	.62
Education (yrs)		16.43 (2.03)	15.73 (2.03)	.17
BMI (kg/m^2^)		25.21 (3.03)	25.97 (4.28)	.32
Hormonal Variables				
Age at menarche		12.83 (1.58)	13.06 (1.57)	.63
Age at menopause		48.17 (4.28)	46.18 (7.15)	.60
No. of reproductive years		34.92 (4.16)	32.22 (7.55)	.39
Natural (vs. surgical) menopause		68.75%	54.84%	.31
Time since menopause (yrs)		9.85 (7.54)	12.81 (8.70)	.38
Time on HT (yrs)		9.40 (6.84)	11.06 (5.97)	.72
Unopposed E (vs. opposed)		38.71%	31.25%	.60
17β estradiol (vs. CEE)		80.65%	53.12%*	.02
Baseline cognitive function				
MMSE		29.17 (.95)	29.35 (.91)	.43
FSIQ		122.23 (8.80)	121.61 (10.02)	.80

Values represent mean (SD) or % as noted.

* p<0.05.

### APOE-ε4 genotype and telomere attrition

APOE-ε4 carriers had significantly greater TL attrition over the 2-year study period than non-carriers ([*F*(1,39) 4.89, *p* = .03] (Figure 1). On average, carriers of the ε4 risk allele lost 435.15 bp more (mean  = −355.21, SEM  = 136.53) than non-carrier controls (mean  = 79.94, SEM  = 134.34). Hierarchical logistic regression modeling adjusting for age, education, baseline TL and HT group revealed that the odds of TL shortening (versus maintenance) over the 2-year study period were more than 6 times higher for APOE-ε4 carriers compared to non-carriers (OR  = 6.26, 95% CI  = 1.02, 38.49; R^2^ = .30 (Cox & Snell); Model χ^2^(1)  = 14.81, p = .011). Controlling for genotype, randomization off HT did not confer increased odds of TL shortening (OR  = 3.97, 95% CI  = 0.59, 26.61) (Table 2).

**Table 2:** Odds ratios and 95% CI for predictors of telomere shortening.

	*Odds Ratio* (*CI*)
Hormone group	
OFF†	
ON	3.97 (0.59, 26.6)
APOE carrier	
NO†	
YES	**6.26** * (1.02, 38.39)

* p<0.05.

† Reference group.

Adjusted for age, education and baseline TL.

Note: R^2^ = .30 (Cox & Snell). Model χ^2^(1)  = 14.81, p = .011.

Non-carrier, Carrier (n = 24, 18) On, Off HT (n = 26, 16).

### Hormone therapy

**Figure 2:**
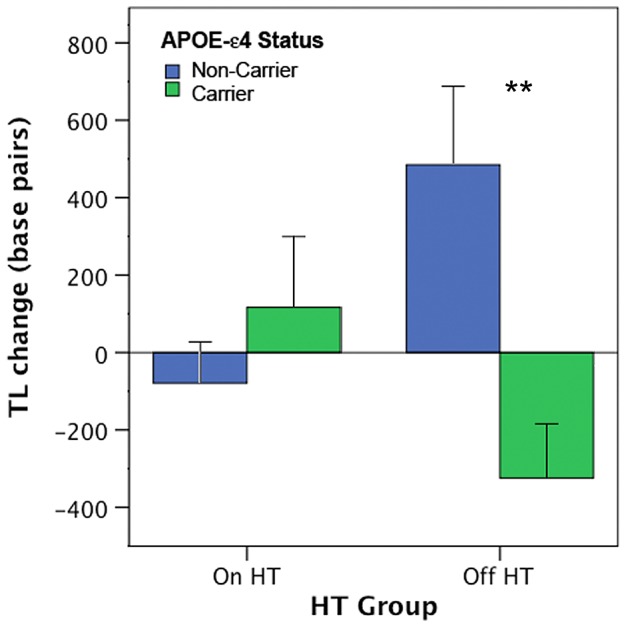
Impact of hormone therapy on telomere dynamics is APOE-dependent. Initial evidence that the impact of the APOE-ε4 risk allele on cell aging is modulated by HT use in mid-life women. Among women who were randomized to stay on HT (n = 26) for the two year study period, no significant difference in TL attrition was detected between ε4-carriers and non-carriers. Among women who went off HT (n = 16), those carrying the risk allele exhibited telomere shortening, while non-carriers exhibited telomere maintenance/growth. Values represent mean TL change (base pairs) adjusted for study covariates. Error bars represent S.E.M, ** p≤.005.

Analysis of covariance revealed an interaction between APOE-ε4 carrier status and HT group on TL change, *F*(1,34)  = 9.17, *p*<.005, effect size *partial η^2^*  = .24 (Figure 2). For participants randomized *off* HT, APOE-ε4 carriers had greater telomere attrition over the two-year period compared to non-carriers, (*p* = .005, effect size *partial η^2^*  = .29). However, for participants who remained *on* HT, no significant difference in TL change between carriers and non-carriers was observed (*p* = .37, effect size *partial η^2^*  = .02). APOE-ε4 carriers who went off HT exhibited telomere attrition across the two-year study period (mean TL shortening  = −321.52 bp). APOE-ε4 carriers who remained on HT showed no evidence of TL decline (mean TL change  = +117.61 bp). Strikingly, there was a strong increase in TL for non-carriers who went off HT (mean TL change  = +490.08 bp). Post-hoc main effects analyses indicated a marginally significant difference in TL change between women who were randomized ON versus OFF HT, for both APOE carrier (p<.05) and non-carrier (p<.05) groups. Further, among participants who remained on HT, there was no difference in TL change between ε4 carriers and non-carrier controls was observed (p>.05). However, for participants who went off their HT regime, a striking difference between carriers and non-carriers was evident (p<.005).

## Discussion

The present longitudinal data demonstrate that the APOE-ε4 risk allele impacts TL dynamics, with ε4 carriers showing significantly greater telomere loss across a 2-year window than matched non-carrier controls. Carriers of the risk allele lost, on average, 435-bp more than non-carriers. Assuming an average telomere loss in adults of 31–63 bp/year [10], [52], [53], the 435-bp shortening observed in APOE-ε4 carriers suggests that their lymphocytes had aged the equivalent of 7–14 years compared to non-carriers. Logistic regression data further demonstrate that the APOE-ε4 allele confers increased odds of leukocyte telomere attrition. The odds of an APOE-ε4 carrier exhibiting telomere shortening over a 2-year period were more than 6 times higher than a non-carrier, adjusting for some established risk factors and potential confounds. Further, the randomized study design provided an opportunity to examine the impact of HT on cell aging. APOE-ε4 carriers who suspended their HT regimen lost an average of 322 base pairs over the two year window, but carriers who remained on HT showed little to no TL decline. Importantly, for non-carriers there was no evidence that HT conferred a protective effect on cell aging.

Strikingly, the impact of the APOE-ε4 risk allele on accelerated cell aging was evident in high functioning, mid-life women who were free of significant medical co-morbidity. This suggests that the impact of the APOE risk allele on telomere attrition begins in the absence of, or prior to, clinical and behavioral evidence of dementia. Short leukocyte TL is an emerging biomarker of biological age and has been associated with a number of chronic and age-related disorders, including depression [54], dementia [12], [16] and early mortality [11], [12], [14], [55], [56]. The present study builds on these findings by demonstrating that a major genetic risk factor for age-related cognitive decline and dementia, APOE-ε4, confers increased odds of premature, accelerated cell aging in currently high-functioning healthy individuals.

Previous cross-sectional studies indicate that APOE-ε4 status is related to TL although the nature of this relationship has been inconsistent, likely given the constraints of a cross-sectional design. Some studies report shorter TL in APOE-ε4 carriers compared to non-carriers [25], but a recent study observed the opposite association [57]. These investigations provide initial evidence of a link between APOE and TL, but their cross-sectional nature limits drawing firm conclusions about the causal relationship between APOE-ε4 and accelerated cell aging. The current longitudinal findings demonstrate that APOE-ε4 carriers exhibit pronounced TL attrition over just a 2-year window and the data suggest that HT, begun at the onset of the menopausal transition, might buffer against cell aging specifically in women at risk for cognitive decline. Importantly, the putative protective actions of HT on telomere dynamics were not observed in APOE-ε4 negative women. For non-carriers, randomization off HT was associated with telomere lengthening. This pattern of telomere growth in a sub-set of individuals is not well understood but is nevertheless consistent with the few but growing number of investigations that have succesfully tracked TL change over time in the same cohort [14], [51], [58], [59]. In one of the first longitudinal TL studies, Epel and colleagues [14] followed a cohort of 236 elderly individuals (mean age 74) over a 2.5-year period and found that 24% showed TL lengthening. The biological significance of telomere elongation over a short time period is unkown and under investigation.

A limitation of the current study is the sample size, which precluded our ability to explore the complex influence of specific HT regimens (e.g. estrogen opposed versus unopposed by progesterone) on TL dynamics. At present, no other longitudinal study has examined the relationship between HT and telomere attrition in vivo in women. Thus the current findings, which suggest a protective effect of HT for APOE-ε4 carriers, should be considered a first step which needs to be replicated in a larger cohort designed to systematically probe the impact of specific HT formulations on TL dynamics. In our sample, although it was too small to examine various types of HT, these were not distributed equally. Approximately 80% of women who suspended HT for the 2-year study were on a hormone regimen that included 17β-estradiol (versus conjugated equine estrogen), compared to 50% of women who remained on HT. Future studies must test whether the putative protective effects of HT for ε4 carriers (and the potential non-protective effects for non-carriers) are attenuated or enhanced across different hormonal regimens.

A growing body of evidence from *in vitro* and *in vivo* animal studies has identified a number of potential pathways by which endogenous estrogen and, subsequently, exogenous hormone therapy could modulate telomere dynamics. Estrogen stimulates hTERT gene expression and potentiates telomerase activity [28]–[32], [60], [61]. Estrogen may also modify TL indirectly given that oxidative stress and chronic inflammatory activity are strong predictors of short TL [1], [2] and estrogen may exhibit anti-inflammatory and anti-oxidant actions [33], [34], [61].

Further studies are critical to clarify the mechanisms by which HT might decelerate telomeric aging in APOE-ε4 carriers but have no protective effect for non-carriers. One clue is that estrogen modifies the expression of the APOE gene through an estrogen receptor (ER) dependent pathway [42]. Apolipoprotein-ε mRNA and protein are up-regulated in response to ERα activation and down-regulated in response to ERβ activation [62], suggesting a potential therapeutic target for differentially regulating APOE expression. Yaffe and colleagues also found evidence of a gene-hormone interaction in a longitudinal study of over 3,000 older women who underwent annual cognitive testing over a six-year follow-up. A significant interaction was found between APOE-ε4 carrier status and use of hormone therapy on long-term cognitive decline [41], [63]. Potential mechanisms through which APOE status may interact with estrogen (both endogenous and exogenous) warrant further investigation. APOE variants have different anti-oxidant profiles, with the ε4 allele showing decreased antioxidant activity relative to ε2 and ε3 alleles [64]. The ε4 allele is also associated with increased accumulation of amyloid-β peptides, whose cytotoxicity is mediated in part by increased oxidative stress [65]. Thus, the accelerated telomeric aging observed in ε4 carriers may be due to increased oxidative stress paired with reduced anti-oxidant capabilities conferred by the ε4 allele.

In sum, this study represents the first longitudinal demonstration of accelerated cell aging in APOE-ε4 carriers. Our finding that even high-functioning, healthy mid-life women with the ε4 allele bear markers of cellular aging suggests the need for further research to understand the potential utility of leukocyte TL as an early indicator of future dementia risk. These data provide strong evidence that the impact of the APOE risk allele on telomere attrition begins in the absence of, or prior to, clinical and behavioral evidence of dementia. Futher, the data provide initial evidence that hormone therapy, begun at the onset of the menopausal transition, might buffer against TL attrition in women at risk of cognitive decline. Importantly, terminating HT had beneficial effects for non-carriers, indicating that HT may have differential effects on cell aging across genotypic subgroups. Going forward, it will be crucial to extend these initial data to a larger epidemiological sample to systematically probe whether specific HT formulations, timing of initiation, and duration of use differentially impact cellular aging.
